# Bursty properties revealed in large-scale brain networks with a point-based method for dynamic functional connectivity

**DOI:** 10.1038/srep39156

**Published:** 2016-12-19

**Authors:** William Hedley Thompson, Peter Fransson

**Affiliations:** 1 Department of Clinical Neuroscience, Karolinska Institutet, Stockholm, Sweden

## Abstract

The brain is organized into large scale spatial networks that can be detected during periods of rest using fMRI. The brain is also a dynamic organ with activity that changes over time. We developed a method and investigated properties where the connections as a function of time are derived and quantified. The point based method (PBM) presented here derives covariance matrices after clustering individual time points based upon their global spatial pattern. This method achieved increased temporal sensitivity, together with temporal network theory, allowed us to study functional integration between resting-state networks. Our results show that functional integrations between two resting-state networks predominately occurs in bursts of activity. This is followed by varying intermittent periods of less connectivity. The described point-based method of dynamic resting-state functional connectivity allows for a detailed and expanded view on the temporal dynamics of resting-state connectivity that provides novel insights into how neuronal information processing is integrated in the human brain at the level of large-scale networks.

The field of dynamic functional connectivity (dFC) in fMRI is still in its relative infancy where different approaches and methods have been proposed to isolate brain fluctuations in connectivity that have long been known to exist in the brain during rest^1^,^2^. There are multiple unresolved issues regarding which methodology is the better one and whether the fluctuations are statistically significant^3^,^4^ and which preprocessing steps that should be taken^5^.

Methods can be split into three approximate classes. The first derive connectivity estimates over a continuous section of the fMRI session, often using a sliding window^6^,^7^,^8^,^9^,^10^ but other methods exist, such as temporal derivatives^11^. The second utilize individual time-points of the data to construct resting state networks^12^,^13^. These methods generally only use a proportion of the data, for example ref. 13 used 15 percent of the data. Recently, this class of methods has recently shown that short spontaneous event occur throughout the fMRI time course^14^. The third class do not require deriving covariance matrices, but instead classifying the fMRI signal in different ways. The most common from this class is temporal independent component analysis^15^.

In this work, we set out to quantify dynamic aspects of neuronal integration between resting state networks. While all approaches to investigate the dynamics of brain connectivity have their own methodological advantages and disadvantages, we aimed to develop a strategy that maximizes the temporal sensitivity, since this would allow us to take advantage of the recent methodological developments within temporal network theory^16^,^17^. Temporal network theory takes into account that connectivity (edges) between different regions of the brain (nodes) may not always be present and may change over time. Analogously, a person’s entire social network can be, and is often, represented as a static connectivity matrix, but then the temporal information of how frequently each person meets is then lost. However, this information can easily be represented in temporal network theory, where connections expressed in time will represent both who is connected and how often they meet. Temporal network theory opens up the possibility to quantify the nature of network integration as it evolves in time. However, to get to this stage, we need a method that is able to compute single time-point connectivity estimates as well as being sensitive to temporal fluctuations in brain connectivity.

Given this background, we optimally want a method for dFC analysis that provides us with an estimate of connectivity that has high temporal sensitivity. To achieve this, we need to sample multiple time-points to get a robust estimate of the signal covariance. These two requirements are difficult to reconcile in a single method. The sliding-window method becomes a trade-off between the two, since increasing the temporal sensitivity (i.e. reducing the window length) will decrease the amount of points used in estimating the covariance, which comes at the cost of a more noisy estimate of the covariance. In this paper we propose the foundation for a point-based method (PBM), a framework that aims to provide high temporal sensitivity without the drawbacks that are inherent to the sliding-window approach. The logic used here is to remove the restraint that the estimates of covariance require time-points to be “neighbors” to each other. Instead we propose to gather time-points to estimate the covariance by identifying clusters that consist of time-points that have a similar global spatial pattern and estimating the covariance for each of these clusters. Thus, the PBM approach clusters time-points into similar spatial patterns and then compute the covariance for each of these clusters.

This framework for dFC analysis was developed to utilize temporal network theory. In this work, we focus on one particular property: bursts. Bursty properties in temporal network theory have been applied to a wide range of social activities^18^,^19^,^20^,^21^. Theoretically, bursty processes are characterized by a higher occurrence of both shorter and longer durations of inter-contact times (ICT, i.e. the time duration between consecutive time-points of connectivity) than what is expected by chance alone (e.g. a Poisson distribution). Consider for example the simple case of a single dynamic, fluctuating degree of edge connectivity between two brain regions. Then, we say that the temporal pattern of connectivity between the two regions is bursty if the instances in time when the two regions are connected (ICTs) are characterized by a fat-tailed distribution where the probability of longer ICTs decays slowly. In contrast, a non-bursty process would either have a completely random presence of edge connectivity (i.e. follow a Poisson distribution), or possess a tonic/periodic connectivity pattern for which a presence of connectivity is either constant or oscillating over time with a small temporal variance in ICT. In this work we show that between-network connectivity has a bursty connectivity profile.

## Results

### Illustration of the derivation of s- and t-graphlets

Our aim is to construct a connectivity matrix per time point and then test whether the temporal pattern of connectivity for an edge is bursty. For this we developed a method which we first illustrate with a simplified example (see methods for technical details).

We use the term *graphlet* to describe multiple connectivity matrices over a non-nodal dimension. The nodal dimension here is spatial (i.e. ROIs/nodes represent different brain regions). The term graphlet is a useful concept when analyzing fluctuations of graph metrics over some variable (see ref. 22 where “f-graphlets” were created expressing network fluctuations over frequency). The method used here creates graphlets over states (s-graphlets) and time (t-graphlets).

**Figure 1:**
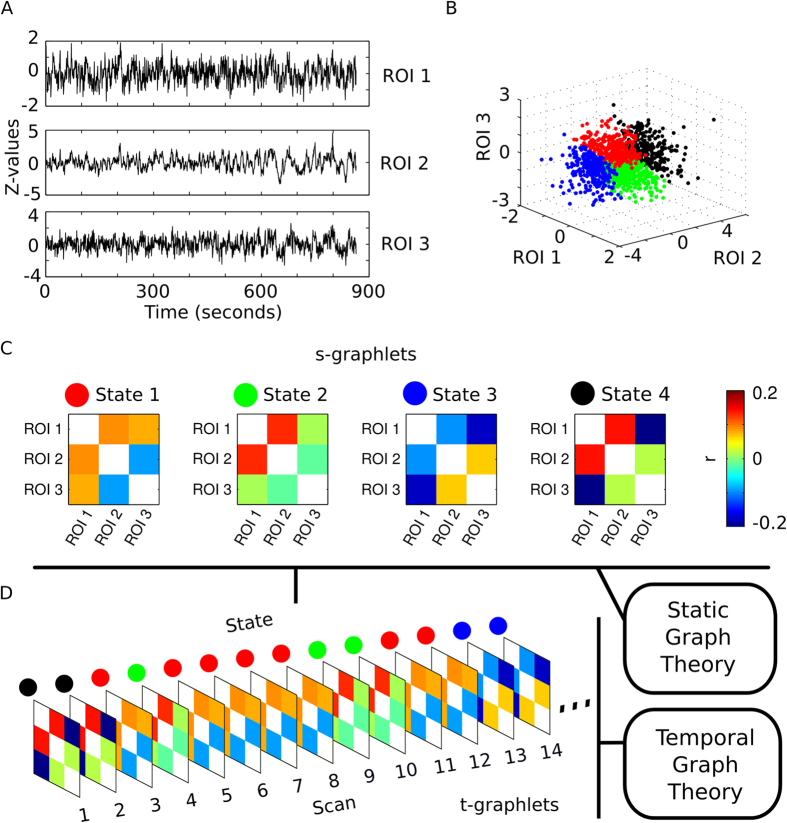
A schematic example that serves to illustrate the methodological framework used in this paper. (**A**) Z-values from three different regions of interest (ROI) during a single resting-state scan (demeaned and normalized by subtracting the mean and dividing by the standard deviation). (**B**) A 3D representation where each time-point is presented as a point in a three-dimensional space where each dimension represents the z-values of each of the three ROIs. Subsequently, k-mean clustering is performed which results in four clusters (color-coded). The individual clusters represent different states based on the underlying similarities in cluster space that is spanned by the time-series extracted from the three ROIs. (**C**) For each cluster derived in the step shown in B, a connectivity matrix between all ROIs is computed by means of a Pearson correlation that is based on all the time-points that belonged to that particular cluster. We name each connectivity matrix a state-graphlet (s-graphlet) on which, if desired, standard (static) graph theoretical measures can be computed. (**D**) In a last step, we create a time-series of s-graphlets where they act as representative connectivity templates at each instance in time. We call the time-series of connectivity matrices temporal graphlets (t-graphlets). Lastly, on the t-graphlets, we apply temporal network theory to compute dynamical properties of functional resting-state connectivity.

In our simple example, we take the time series extracted from three ROIs in a single subject after preprocessing (Fig. 1A). The time-points can be grouped by clustering time points along the spatial dimensions using the k-means clustering method (*k* = 4) (Fig. 1B). We name the clusters “states”, which implies that each cluster contains time-points that have similar relative global configurations of the signal intensity between the ROIs. For each state, a connectivity matrix is created that is based on the covariance of all the time-points clustered in that state (Fig. 1C). This gives *k* number of unique connectivity matrices. The corresponding connectivity matrix for a state is called an s-graphlet. Clearly, the connectivity profiles among the four s-graphlets shown in Fig. 1C are different from each other. Of note, conventional network theory measures can be applied on s-graphlets (e.g. degree centrality, betweenness centrality and community detection, see ref. 23). Finally, a connectivity matrix is assigned to each time-point by applying a function on the derived s-graphlets. In this work, we use a simple t-graphlet derivation function where each time-point is identical to the s-graphlet corresponding to the cluster the time point was a part of (Fig. 1D, see also Supplementary Figure S1 for an illustrative example). With a time series of connectivity values, methods from temporal network theory can be applied (e.g. burstiness, persistency, temporal motifs, see ref. 16). Different strategies to derive t-graphlets from s-graphlets will each bring with them their own assumptions, advantages and disadvantages. The one used here is simple and utilizes only similar assumptions from conventional functional connectivity studies, but limits the span of the time series to *k* possible values (see discussion).

### Results of k-mean clustering the spatial dimensions

**Figure 2:**
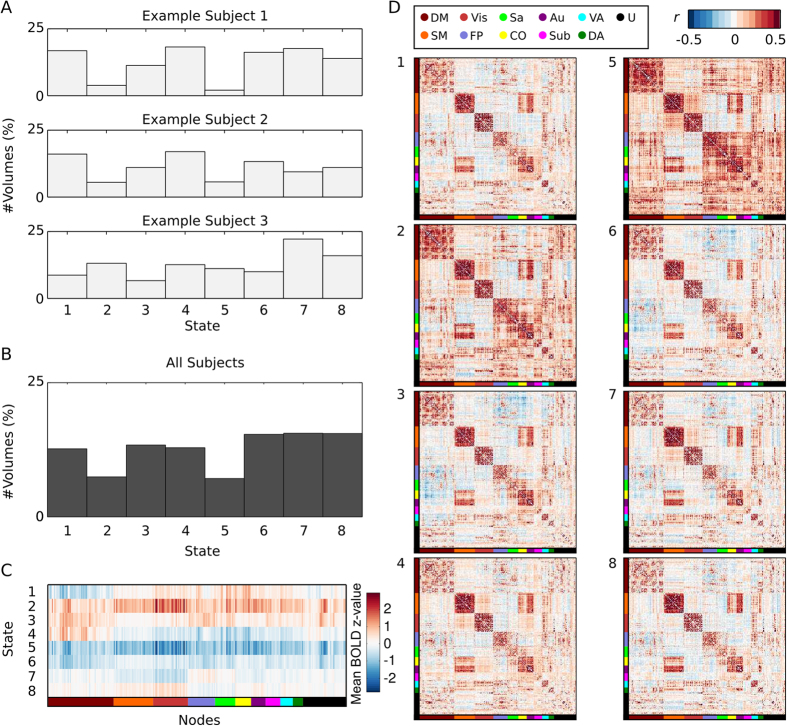
Distribution of image volumes (or equivalently, time-points) across states (s-graphlets) and subjects. (**A**) Percentage of image volumes in three representative subjects that were assigned to each of the eight states by means of k-mean clustering (number of clusters, k = 8). (**B**) Percentage of image volumes for all subjects that were assigned to each of the eight states. (**C**) Average Z-value over all assigned time-points in each state for each node. The colored bar at the bottom of the figure corresponds to the nodes assigned to each network. (**D**) Connectivity matrices for all states/s-graphlets using time-points concatenated across all subjects. The nodes in each connectivity matrix are ordered according to their designated resting-state network (DM - default mode, Vis - visual, Sa - Saliency, Au - Auditory, VA - ventral attention, SM - sensorimotor, FP - fronto-parietal attention, CO - cingulo-opercular, Sub - Subcortical, DA - dorsal attention, U - unassigned).

We leave our illustrative example and turn our attention to the full dataset (100 subjects from the Human Connectome Project^24^, resting-state fMRI, 1200 time-points, 264 spatial dimensions, reduced to 67 spatial dimensions with PCA, 10 predefined networks). K-means clustering of time points along the spatial dimensions (k = 8) resulted in 8 different clusters. States identified were found across subjects (Fig. 2A). Some states generally occurred more frequently than others (Fig. 2B).

**Figure 3:**
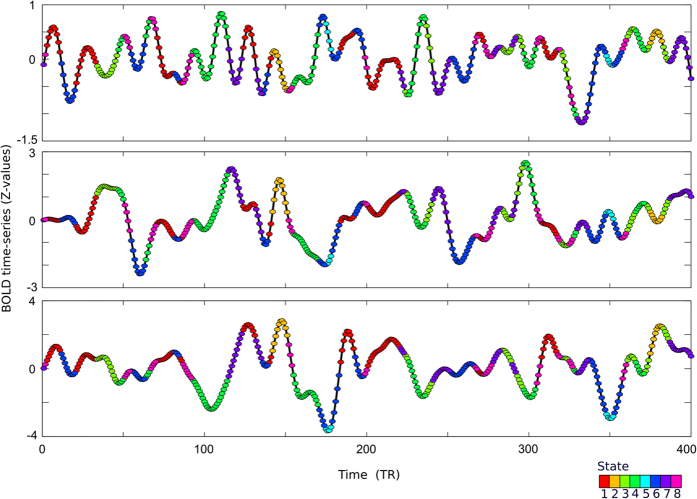
Assignment of states for resting-state fMRI data where state (color-coded) is plotted on top of the standardized BOLD time-series for three different ROIs in one representative subject (only the first 400 time-points are shown). Each BOLD signal intensity time-point is assigned to one of the eight possible states by the point-based method. Here, it is important to point out the point-based clustering method does not result in a trivial partitioning of the time-series based on the amplitude of the BOLD signal. For an example, the first peak (t = 10 TR) in the first ROI (upper panel) with a Z-value greater than 0.5 is assigned to state 1 whereas a trough at t = 204 TR (Z-value less than −0.5) is also assigned to state 1. Many other examples of this behavior can be found in Fig. 3, for example the second ROI (middle panel) has local trough at t = 240 TR and a peak at t = 300 TR that are assigned to the same state (state 3). Hence, the point-based method yields a complex pattern of states that are based on the global features of the space spanned by the ROI signal intensity time-courses using the PCA components as input.

The clusters were derived from the BOLD time series of ROIs and it is important to consider how the amplitude of signal varies between states. The average BOLD z-values for each ROI are shown in Fig. 2C for each of the eight derived states. From Fig. 2C, some trends can be observed. For example, in the slightly less occurring states (states 2 and 5, see Fig. 2A), all ROIs have their lowest and highest amplitudes respectively. Moreover, for state 4, the nodes/ROIs residing in the default mode network have on average higher BOLD amplitude than all other nodes. Caution is however needed in interpreting the amplitudes shown in Fig. 2C. It is important to keep in mind that clustering of the BOLD amplitude of ROI time series is based on the global spatial context (i.e. the relation to all 264 ROIs), not merely the signal intensity of one ROI. This claim is justified when considering the variance of the amplitude of ROI signal intensity time series (Fig. 3). The behavior exemplified in Fig. 3 shows similar intensities of a single time series being assigned to multiple different states, demonstrating that clustering is based on the relative global configuration between the ROIs.

One final aspect regarding the clustering performance that might be of concern is what is happening when a state transition occurs. Two options are possible: Either the activity jumps in some non-linear fashion or that there is a gradual transition between states. By taking the average Euclidean distance of the signal intensity of all ROIs at successive time points preceding and following a state transition, we can observe that the largest difference occurs at the transition between states (Supplementary Figure S4). However, this distance between successive time points increases as a state transition approaches. This suggests that the dynamic evolution of the BOLD signal is a gradual change between states rather than an abrupt, nonlinear transitions. That being said, there is still a large difference between time points when there is a state change, entailing that a larger change occur at a point of state transition, justifying their classification.

### Significant differences in s-graphlet connectivity

For each state/s-graphlet, all time-points that belonged to the corresponding cluster were used to compute the connectivity matrices between all ROIs as shown in Fig. 2D (see also Supplementary Figure S5 for a Kamada-Kwai spring-embedded plot of all states). The two s-graphlets with the least amount of time-points/image volumes attached to them (s-graphlets 2 and 5) show very visible increases in overall brain connectivity. Other differences between s-graphlets in Fig. 2D, although less apparent, can be observed. These differences can be see near both the diagonal, indicating differences in within-RSN connectivity, and away from the diagonal, indicating an increased degree of between-RSN connectivity.

The question whether the derived connectivity matrices for the different states are actually significant differences in functional connectivity is of importance. As noted in our simple example shown in Fig. 1, there is no built-in mechanism in the k-means clustering method that enforces s-graphlets/state connectivity matrices to be significantly different from each other. Hypothetically, the variance along the spatial dimensions for each state could be very similar between two clusters, which would result in s-graphlets that do not significantly differ from each other. To ensure that this was not the case, we tested for significant differences between all the s-graphlets using a data shuffling method (p < 0.001, two-tailed, FDR-adjusted).

**Figure 4:**
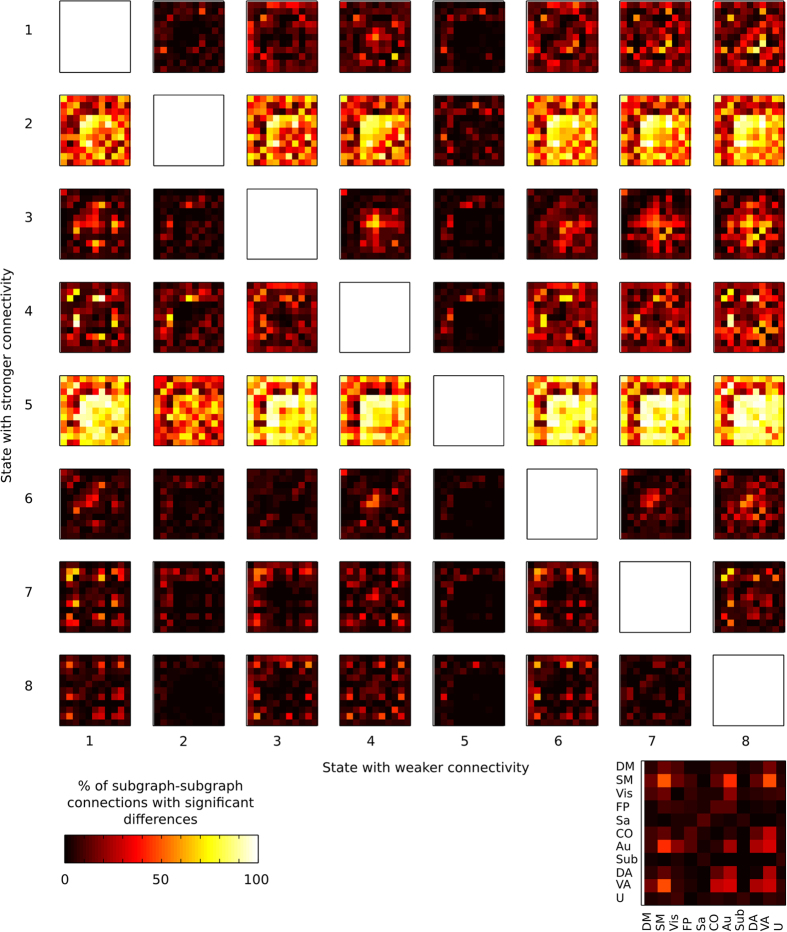
Difference in connectivity between s-graphlets at the level of resting-state networks. The results show that all s-graphlets have significant differences between resting state networks. Percent of edges between different resting state networks for each s-graphlet comparison where an edge was significantly larger in one state compared to the other (p 0.001, FDR adjusted).

Since the focus of our study was to examine the dynamics of network integration, we have chosen to present how the s-graphlets significantly differed from each other at the level of resting-state networks. A comparison of the proportion of varying edges of connectivity for all pair-wise combinations of resting-state networks for all eight s-graphlets is shown in Fig. 4. Each matrix shows the percentage of edges that is significantly larger in one s-graphlet compared to the other (same test as above). Taken together, the results shown in Figs 3 and 4 strongly suggest that the k-mean clustering of the resting-state fMRI data into 8 states/s-graphlets have yielded a partitioning that separates the data into states that each have their own unique characteristics in terms of connectivity profiles, both at the level of individual edges between ROIs as well as for the ten a priori defined resting-state networks.

### S-graphlets do not correlate with movement

There is a possibility that at least one of the states/s-graphlets correlates with movement, as micro head-movement is known to be a major concern for resting state connectivity studies^25^,^26^. The relative amount of motion-afflicted time-points for each state/s-graphlet is displayed in Supplementary Figure S6. Although motion-afflicted time-points are present in all states, it can be seen that state 8, and to some small extent also states 2 and 7, had a slightly higher disposition for micro head-movement in relative measures compared to the other five states. So the question is if the relatively higher propensity for head-movement observed for state 8 should be taken as an indication it is a state that can be attributed to micro head-movements? When considering this possibility it is important to keep in mind that the numbers of motion-afflicted time-points provided in Supplementary Figure S6A are given as a percentage of the total number of time-points across subjects that had a FD-value higher than 0.5. Importantly, the average (across subjects) number of time-points for which the FD-value was larger than the chosen threshold was 14.6 time-points per imaging session (1.2 percent of the total of 1200 time-points). For example, this implies that the number of approximately 330 image volumes deemed to be affected by micro-motion in state 8 as shown in Figure S6B corresponds, on average, to 4.32 time-points afflicted with micro head-motion per session. So, although the amount of motion-afflicted time-points differs between states, the difference in absolute terms is quite small, which suggests that no particular state can be singled out as a state that is driven by micro head-movements.

### Temporal sensitivity and state transitions

**Figure 5:**
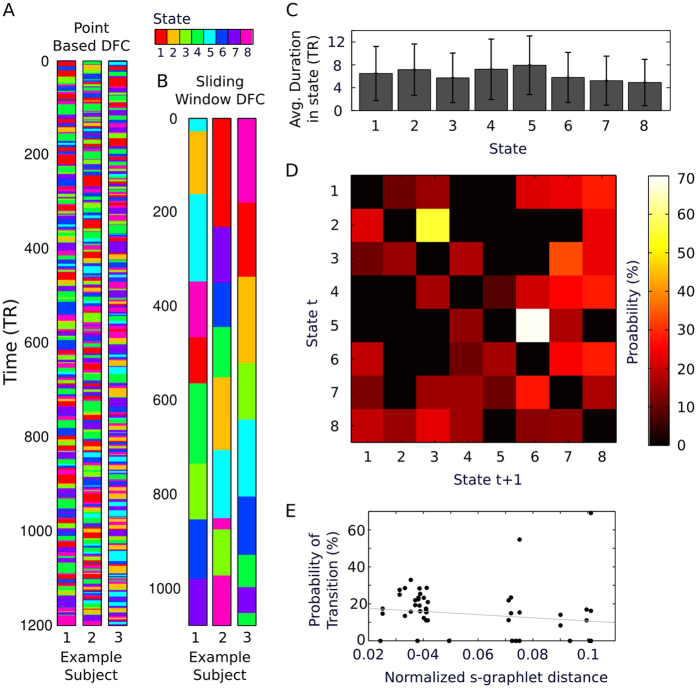
Transition of state in resting-state fMRI time-series. (**A**) Example of the temporal transition of s-graphlet/state in three representative subjects (time is here expressed in TR (scans) = 0.72 seconds) using the point-based method of dynamic functional connectivity. (**B**) Corresponding temporal transition of state in the same subjects using the sliding window method (window length = 86.4 seconds). Note that although the coloring scheme used is the same for the two methods shown in panels A and B, this does not imply that the actual state connectivity patterns are identical. (**C**) The average (across subjects) duration (again expressed in TR) a subject stays in any given state (1–8) before a transition of state occur. Error bars depict the standard deviation. (**D**) Transition probability graph showing the probability that a transition of state occur between time t and t + 1. Each matrix row adds up to 100 percent. (**E**) The probability of the occurrence of a state transition versus the distance between two s-graphlets. The figure shows a significant negative correlation between the two (*ρ* = −0.3338, p = 0.0119).

The state assignment through time show quite quick transitions in three example subjects (Fig. 5A). For visual comparison, we performed a sliding window dynamic connectivity analysis with the same subjects (Fig. 5B). Note, the comparison with the sliding window analysis is illustrative of the different temporal scales of the two methods, but not quantitative in terms of the “states” identified by the methods and the sliding window method does not necessarily require identification of states. Rather, with this qualitative illustration, we merely wish to show that PBM is more appropriate to investigate quicker temporal properties.

The average duration for staying in a given state/s-graphlet is 6.3 scans or 4.5 seconds (Fig. 5C). Histograms that shows the full distribution of the duration for staying in each state is shown in Supplementary Figure S7.

We then computed the transition probabilities between different states. The state transition probability matrix shown in Fig. 5D reveals that some states are more reachable than others. It is noteworthy that the two states that show an apparent high degree of connectivity across RSNs (s-graphlets 2 and 5, see Fig. 2C) are both highly likely to transfer into states 3 and 6 respectively, for which the global pattern of strength of connectivity for the visual, auditory, and motor networks are stronger in states 2 and 5 whereas the frontal-parietal and default mode networks harbor the opposite pattern. Moreover, we asked whether the probability of transitioning from a state to another correlated with the similarity between the corresponding s-graphlets. Using the normalized taxicab distance, we found that there is a negative correlation between the probability of transitioning from one state to another and the corresponding similarity in terms of distance between the two s-graphlets (Fig. 5E, *ρ* = −0.3338, p = 0.0119), which speaks against the possibility that states are too short and that state transitions are abrupt and non-linear.

### Bursty temporal properties of connectivity

To explore the temporal network properties, an s-graphlet is assigned to each time point. The s-graphlet assigned is that which was derived from the cluster that the time point was assigned to (see example shown in Fig. 1D). The series of connectivity matrices now express temporal information, becoming a t-graphlet, and thus allowing us to study how the differences in connectivity evolve in time. The t-graphlets were then converted into binary connectivity matrices (propertional threshold set at 5% and 10%).

**Figure 6:**
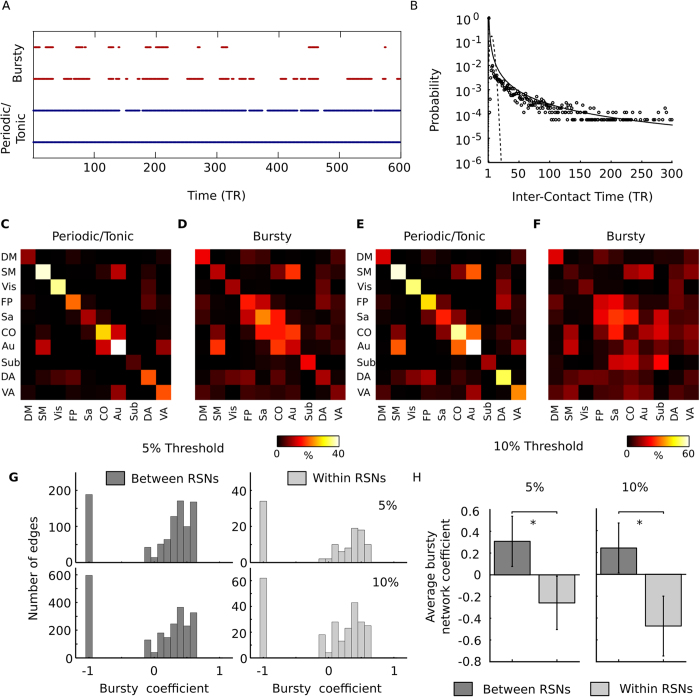
The burstiness of between- and within-resting-state brain connectivity. (**A**) An example of two bursty (red) and two periodic edges (blue) extracted from one representative subject while retaining the top 5 percent of the strongest edges (only the first half of the fMRI session is shown). Line indicates the presence of the edge. Note that periodic edges of connectivity can always be present. (**B**) An example of the distribution of inter-contact times for a single edge of brain connectivity. Data from all subjects were included and the graph was cropped to show the inter-contact times up to 300 TRs (216 seconds). The dashed line shows a fitted Poisson distribution and the solid line shows an approximate Pareto distribution (*T*^−*α*^) with *α* set to 1.8 and T is the inter-contact times. The bursty nature of brain connectivity is evident by the fact that the distribution of inter-contact times displays a heavy fat-tail with a varying degree of longer inter-contact time as well as a cluster of very short inter-contact times. This behavior is in stark contrast to the corresponding Poisson distribution. (**C**) Percentage of edges between each combination of resting-state networks that had a periodic temporal pattern (p ≤ 0.05, two tailed). Note that the diagonal elements (within RSN-connectivity) had the majority of the periodic edges. (**D**) Percentage of edges between each RSN combination that had bursty edges (p ≤ 0.05, two tailed) when keeping the top 5% of all edges. Note that the between-RSN integration shows a large presence of bursty connectivity. (**E**) Same as C but keeping the top 10% of all edges. (**F**) Same as D but keeping the top 10 of all edges. (**G**) Distribution of burstiness coefficient over all edges for within and between networks for 5% and 10% threshold. (**H**) Average burstiness coefficient for all between-RSN edges and within-RSN edges when withholding the top 5% and top 10% of all edges (edges must be present in at least one time-point) showing a statistical difference between the two (p 0.05). Error-bars show standard deviation.

We then tested whether the temporal pattern of connectivity was bursty, random or non-bursty. Non-bursty edges will have either tonic or periodic activation profiles. Edge examples of bursty and periodic/tonic inter-contact times (ICT) from a single subject are given in Fig. 6A. The empirical distribution of the ICT obtained from a single edge (pooled over subjects) was compared to a fitted Poisson and an approximated Pareto distribution (Fig. 6B). The ICT distribution shows a fat-tail, indicative of a bursty process.

We calculated the percentage of edges that showed either significantly bursty or periodic/tonic temporal patterns of connectivity (p ≤ 0.05, two-tailed). At both edge thresholds used (top 5 and 10 percent respectively), the results shown in Fig. 6C–F suggest that within-RSN connectivity (diagonal elements) contains a mixture of both periodic and bursty dynamic connectivity patterns. Perhaps rather unsurprisingly, a substantial part of within-RSN temporal connectivity, in particular so for the lower 10 percent edge threshold, is characterized by tonic patterns of connectivity. Although the between-RSN connectivity profiles also display a mixture of both tonic/periodic and bursty edges, the results shown in Fig. 6C–F suggest that between-network integration is dominated by bursty ICTs rather than tonic/periodic ICTs. The distribution of all bursty coefficients are shown in Fig. 6G. Here we see that many edges always remain below the magnitude threshold and get a value of −1 (regardless of threshold choice) showing that they have tonic activation instead of periodic. Then there is a distribution of edges that is, for the most part, above 0 and show, to a varying degree, a more bursty connectivity pattern. By taking the average of the burstiness coefficient for all between-RSN edges and within-RSN edges (where an edge is present in at least one state) demonstrates that the between-RSN edges have a bursty coefficient (on average) and the within-RSNs edges, on the other hand, have a tonic/periodic coefficient (see Fig. 6H). For both edge thresholds (top 5 and 10 percent), there was a significant increase for the average between-RSN burstiness coefficient compared to the average within-RSN burstiness coefficient (p < 0.05).

The results shown in Fig. 6 support the view that the large-scale integration and the flow of information between resting-state networks are largely implemented in the form of bursts, i.e. an absence of connectivity for periods of time that are followed by bursts of activity during which an exchange in information between resting-state networks occurs. A possible concern is whether the bursty pattern was induced by the choice of k for k-means clustering. This concern is unwarranted, since a similar pattern of burstiness for between-network connectivity can be found when other k values are used (k = 5 and 12, see Supplementary Figure S8).

## Discussion

The use of temporal network theory combined with the point-based approach to investigate dynamic resting-state fMRI connectivity has allowed us to gain new insights into dynamic brain connectivity. In particular, we have shown bursts in large-scale brain connectivity between resting state networks. However, the question whether the bursty activity identified in this work can be correlated with the underlying neuronal activity is still an open question that should be target in more detail in future research. Further, we have presented a novel framework for dFC, PBM, that in part builds upon previous methods^12^,^13^. However, several features of the proposed method make it suitable for investigating the temporal properties of resting-state fMRI data (e.g. incorporating the whole time series in the analysis). The fact that each correlation matrix at any time-point is not dependent on its immediate neighbors, which allows for a greater temporal sensitivity in the analysis and does not require a window length parameter (but does require parameters for the chosen clustering method). Furthermore, we have shown that the fluctuating states have significantly different connectivity profiles, a property which the sliding window method has difficulties to achieve within one session^4^.

A considerable amount of resting state network analysis have focused on isolating different networks that presumably to some degree reflect different cognitive processes in the brain^27^. However, information still needs to be passed on between RSNs at some time-point. In recent years there has been an increased interest to study the dynamic nature of the brain’s connections (which has been referred to the “dynome”^28^). The dynamics of between-RSN connectivity as revealed in the present work provides a rationale for why previous studies on resting-state fMRI unequivocally have found well segregated RSN but far less information regarding between-network integration, since its bursty nature would make it harder to detect in static connectivity studies.

One outstanding question is whether the bursts seen on a large-scale network level correspond to the bursty behaviour of neuronal spiking. It is well known that the BOLD is a neurovascular event that is rather sluggish in time and this might cast doubts on whether the rather fast transitions between states shown here are indeed feasible or reflect neuronal activity. It is an outstanding question whether, when a collection of single neurons display a bursting pattern of activity, and they repeatedly fire discrete groups (or bursts) of spikes, that this scales up to bursty connectivity that can be identified with fMRI. Further research is needed to understand the precise electophysiological correlates of the bursts isolated here from the BOLD signal. However, there is ample evidence that electrophysiological changes detected with EEG correlate with the BOLD signal^12^,^29^,^30^,^31^,^32^,^33^,^34^. Electrophysiological investigations of the temporal properties of brain signals have found fat tail/power law distributions in both EEG^30^ and MEG^35^,^36^,^37^ data which lends credence to the idea that similar temporal properties prevail in fMRI data.

There are several limitations and considerations that needs to be discussed in relation to the proposed method. First, we would like to emphasize the fact that any type of clustering algorithm can be used for the point-based method, but whichever clustering technique that is used, it bears its own disadvantages as well as advantages. We opted to use the k-means algorithm mainly due to its previous use on fMRI data and its simplicity. However, other methods are possible and we are noncommittal regarding the choice of k-mean used in PBM. We have shown that the global spatial dimensions are indeed being clustered but it remains to be shown which clustering technique that performs optimally in terms of detecting fluctuating connectivity between networks. It is possible that clustering on more local features (e.g. taking nodes from just two RSNs) will better isolate dynamic features between these networks than considering the entire brain context. We, at present, consider the global clustering a preferable strategy as it allows for the possibility of multiple networks integrating information (e.g. presumably, there should be a difference when the auditory and attention networks cooperate and when the visual, auditory and attention networks cooperate), but until a rigorous comparison of clustering the spatial dimensions is performed, it is difficult to provide a definite answer to this question. However, regardless which clustering method that turns out that have optimal performance, it can be substituted into the PBM framework presented here.

When the derived states transitioned we found a larger difference between the ROIs at successive time points. This is in line of slight non-linearities found in previous research behind state switches^38^.

Moreover, the cluster parameter needs to be selected and we believe that our choice of k = 8 is rather conservative and well in line with previous investigations of dynamic resting-state functional connectivity^10^,^13^. Yet our overall findings of bursty mode of between-network connectivity together with a largely period/tonic mode of within-network communication were consistent for a wide range of choices of k (k = 5 and 12, see Supplementary Figure S8). Further, our results could be reproduced in an independent dataset (see Supplementary Methods and Supplementary Figure S3). Taken together, we feel confident that our findings are not critically dependent on cluster dimensionality. There is a valid concern regarding our thresholding strategy used. It is possible that choosing a variance-based threshold over a magnitude based threshold prior to calculating the bursty coefficient could reveal more dynamics of the within-network connectivity^39^.

The creation of t-graphlets by only using corresponding s-graphlets into a time series limits the temporal variance in connectivity to the number of s-graphlets that exist. The question remains whether this mapping is reasonable? Our mapping scheme is fully compatible with the general underlying assumptions of functional connectivity. We assume that an interaction between brain regions can be inferred from their degree of co-variance and that by splitting the entire dataset into clusters based on their global context of brain connectivity, we can infer the variability of interaction at each of the states. We then add that each time-point is representative of the state it belongs too. Could this derivation of t-graphlets induce the bursty properties detected? In the worst case, this method may increase the number of shorter inter-contact times if the connectivity changes considerably during the, on average, 6.3 time points when a state persists. But it would not effect the longer inter-contact time points as each occurrence of a state should contain at least one volume (time point) prototypical of that state. This entails that the general distribution would still be preserved.

While the analysis pipeline presented here is a simple mapping procedure to illustrate the conceptual idea of the PBM framework, more complex methods of creating t-graphlets based on the clustering information and s-graphlets is indeed possible (e.g. deriving t-graphlets based on the weighting of s-graphlets from the distance from cluster centroids). Finally, while we have only investigated the property the burstiness of dynamic connectivity, there is a wealth of possible measures from temporal network theory which can be applied.

In sum, we have presented a novel point-based method (PBM) that takes full advantage of the resolution provided by low TR resting-state fMRI. In doing so we have shown that measures derived from the theory of temporal graphs can be successfully applied to resting-state fMRI data. This has resulted in new insights regarding how functional integration between previously well-known and segregated resting-state networks occurs in time. The results from the point-based dFC method suggests that the functional integration between resting-state networks has a unique temporal pattern during resting-state conditions that encompasses short bursty periods of connectivity. We believe that the presented method holds promise to be used as a versatile tool to quantify dynamic network integration between segregated networks during different experimental conditions.

## Methods

### Data used, regions and subgraphs of interest, pre and post-processing

The 100 subject dataset from the Human Connectome 500 subject release was used^24^,^40^. We used the first resting state session RL (i.e. phase-encoding gradient in the right-left direction). Further information regarding the MR acquisition parameters can be found in refs 24 and 41. The data had undergone image preprocessing and removal of artifacts from non-neuronal origins by means of the FIX ICA (FMRIB’s Independent Component Analysis-based X-noisifier) data artifact rejection process which removed ICA components from the data that were considered to constitute signal contributions from white matter, cerebro-spinal fluid, head movement, cardiac and respiratory sources^40^,^42^,^43^,^44^). The data consisted of 1200 time-points per subject (TR = 0.72).

264 spherical Region-of-Interests (ROI) with a radius of 10 mm defined along cortex and sub-cortical nuclei was used (with the centers taken from ref. 45). Each ROI was assigned to a resting state network (as described in ref. 46). The 10 resting-state network definitions used were: DM–default mode (58 nodes), SM–sensorimotor (35 nodes), Vis–visual (31 nodes), FP–fronto-parietal attention (24 nodes), Sa–saliency (18 nodes), CO–cingulo-opercular (14 nodes), Au–auditory (13 nodes), Sub–cortical network (13 nodes), DA–dorsal attention (11 nodes), VA-ventral attention network (9 nodes) and unassigned (38 nodes). The layout of the nodes is shown in Supplementary Figure S2.

To minimize the effect of movement^25^,^26^, “scrubbing” was performed. The framewise displacement (FD) for each image volume was computed (rejection at FD > 0.5). Rejected volumes were deleted and missing data estimated using a cubic spline interpolation. On average, 1.2% of volumes were rejected per subject. A band-pass filter (0.01–0.1 Hz) was applied, typical in resting-state fMRI studies. time series were transformed into Z-values by subtracting the mean and dividing by the standard deviation. The time series were concatenated across subjects along the temporal dimension before clustering.

### A point-based method to study dFC

We applied a Principal component analysis (PCA) to the fMRI ROI signal intensity time series which reduced the spatial dimensionality of the data to 67 dimensions, which accounted for 85% of the variance. This dimensionality reduction was done to improve clustering performance. The choice of 85% is relatively arbitrary and was chosen due to a trade off between keeping a large amount of the original data and computational time.

K-mean clustering was performed with k set between 2 and 19, where k is the number of clusters the data is split into. For each value of k, we performed up to 1000 iterations to reach convergence, and each choice of k was repeated 20 times to overcome possible initiation differences. K-mean clustering requires a choice of the parameter k. We opted for k = 8, a choice that resonates well with the choices made in previous work that have used clustering techniques to investigate dynamic fMRI connectivity^10^,^13^. Our justification for the choice of k was justified using a separate dataset for which similar spatial patterns were obtained (see Supplementary Information). To further minimize the possibility that our results are influenced by k, key results were reproduced using other choices of k (k = 5 and 12).

### Creating s-graphlets and t-graphlets

*State-graphlets* (s-graphlets) express the connectivity patterns in each of the k states. They were constructed by taking the Pearson correlation coefficient between each of the 264 × 264 ROI pairs for time-points that belonged to each cluster. This procedure yielded 8 s-graphlets and as per convention with the chosen ROI template, the correlation between ROIs with centers less than 15 mm was set to 0, a step taken to prevent an abundance of local connectivity^45^.

To test for significant differences in connectivity among s-graphlets, the null hypothesis was “there was no difference in the connectivity between the states derived through the k-means clustering”. For each cluster pair comparison, a non-parametric Monte Carlo test shuffled time point membership between the clusters, preserving the number of points as in the original clusters. At each permutation, the differences was created by deriving two s-graphlets and subtracting one from the other. After 10,000 permutations, each an edge between two s-graphlets was then compared to its distribution of differences (p < 0.001, two tailed, FDR-adjusted for all edges and s-graphlet comparisons).

*Temporal-graphlets* (t-graphlets) were subsequently constructed as a time series of connectivity matrices for which each time-point was assigned to a s-graphlet, see also the illustration given in Fig. 1. The assignment of a s-graphlet to a particular t-graphlet was dependent on which cluster that time-point belonged to in the clustering step. For each subject, 1200 t-graphlets were created. Each edge in a t-graphlet has k possible values that it can take.

### Transition Probabilities

The probabilities of transition from one state to another were derived from each subject’s time series of connectivity matrices, i.e. their time series of t-graphlets. Only time-points when a transition between states/s-graphlets occurred were considered.

### Sliding window analysis

To qualitatively compare the temporal sensitivity of our method, we contrasted it with dynamic connectivity measured using the sliding window method. For this comparison, the same data and post-processing steps were used. The covariance between each ROI was computed, for each subject using a window length of 120 time-points (86.4 seconds), comparable to the window lengths used in the sliding window literature (see refs 33 and 47). Next, the time window was slid one time point down and a new connectivity matrix was computed. This continued until the end of the time series. We then classified the sliding window connectivity estimates into states with k-mean clustering, which was applied to each subject (k = 8 and followed the same procedure as above). Since the dimensionality was large, the dimensions were reduced using PCA prior to the clustering. 30 spatial components were retained, which accounted for more than 95% of the variance.

### Computing Burstiness

Burstiness measures whether the presence of network edges are characterized by an occurrence of very brief inter-contact times (ICT) intertwined with longer varying ICTs. For a given edge in a network, the inter-contact time is defined as the time duration between consecutive presences. To calculate the ICTs, we first thresholded and binarized the t-graphlets. We only included edges that displayed larger connectivity. A proportion of the highest edges in a t-graphlet were kept. To increase the robustness of our results, we calculated using a threshold of top 5% and 10% of all edges per t-graphlet. An edge’s ICTs were calculated by counting the temporal distance (in samples) between the presence of an edge’s connections. As a final step, the ICTs for an edge were pooled over subjects. The bursty coefficient used here was taken from Goh & Barabási^19^,^16^ and is calculated by:


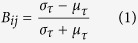


where *σ*_*τ*_, and *μ*_*τ*_ are the standard deviation and the mean of the ICTs, respectively. Burstiness is calculated for each edge *ij. B*_*ij*_’ s value ranges between −1 and 1. When *B*_*ij*_ > 0, it indicates a bursty pattern. *B*_*ij*_ < 0 implies a more tonic/periodic pattern of brain connectivity with less variability in ICTs. Values centered at 0 suggest that connectivity occurs randomly over time.

Statistical tests for burstiness used Monte Carlo non-parametric tests to shuffle the data, which is one of the standard practices of creating null models in temporal network theory^16^. To test for significant burstiness, our null hypothesis was: “the distribution of ICTs followed a Poisson distribution that contained neither tonic/periodic nor bursty ICTs”. A distribution from 200 permutations was created where the temporal order of the thresholded t-graphlets was shuffled to create new ICTs for each permutation. This was done for all edges. To circumnavigate for the multiple comparison problem, we used the maximum and minimum permuted value (taken over all edges), to compute distributions of ICTs for which the empirical values could be compared against. For an edge to be considered bursty, its value had to be above 0 and equal or higher than the 195th highest permuted value from the maximum distribution (p ≤ 0.05, two-tailed). For an edge to be considered periodic/tonic, it had to have a value of less than 0 and equal or lower than the 5th lowest permuted value from the minimum distribution (p ≤ 0.05, two-tailed). We used a significance threshold of p ≤ 0.05 due to the possibility of all permuted values and the empirical values being −1 (i.e. the edge is always present). The choice of 200 permutations was due to computational restraints.

Finally, we tested whether there was a difference in the bursty coefficient for between-RSN versus within-RSN edges. Here, the null hypothesis was “there was no difference in the average between-RSN and within-RSN bursty coefficients”. To test this, a non-parametric permutation test was created by shuffling whether an edge was designated as “within” or “between” RSNs, preserving the number of edges in both groups. The empirical difference between average between-RSN and within-RSN values was compared to the distribution of the differences after 1000 permutations. Values larger than the 975th highest permuted value was classed as significant (p < 0.05, two tailed).

## Additional Information

**How to cite this article:** Thompson, W. H. and Fransson, P. Bursty properties revealed in large-scale brain networks with a point-based method for dynamic functional connectivity. *Sci. Rep.* **6**, 39156; doi: 10.1038/srep39156 (2016).

**Publisher's note:** Springer Nature remains neutral with regard to jurisdictional claims in published maps and institutional affiliations.

## Supplementary Material

Supplementary Methods and Figures
