# Supplementary material for: Bursty properties revealed in large-scale brain networks with a point-based method for dynamic functional connectivity
Source: Sci Rep. 2016 Dec 19;6:39156. doi: 10.1038/srep39156 (PMC5171789; doi:10.1038/srep39156)
Supplement: Supplementary Methods and Figures [file srep39156-s1.pdf]

Supplementary material

Bursty properties revealed in large-scale brain networks with a point-based method for dynamic functional connectivity.

William Hedley Thompson & Peter Fransson

Supplementary Methods

Justifying the number of chosen clusters (k) using an independent dataset.

It first needs to be said that in any type of clustering analysis performed on fMRI data when the number of clusters (k) has to be empirically determined, there are two problems which might occur. (1) The value of k is set too low, resulting in a too small number of clusters being defined which would potentially miss out on some aspects of the brain dynamics. (2) The value of k is set too high which results in too many clusters being defined, leading to an "over-splitting" of the fMRI data. The case of over-splitting could prove to be highly detrimental, as an additional split of a cluster could possible result in that the magnitude of correlation between the BOLD signal intensity values in a cluster switch from being positive to negative (or vice versa), inducing spurious connectivity. Thus, providing as solid experimental support as possible for the chosen value of k is of paramount interest. As an alternate route, the analysis can be extended to include a validation of the results for a range of values of k to show a robustness of an effect that is largely independent on the size of k.

In this work we have both developed a strategy that includes a separate, independent data to validate our choice of k as well as validating our results for different choices of k. With respect to the first approach, we performed additional analyses on the second dataset from the human connectome dataset (i.e. the so called "LR" dataset from the first resting scan in which the direction of the phase encoding gradient (left-right versus right-left) was flipped with respect to the "RL dataset" used in the main analysis, see Ugurbil et al., 2013 and van Essen et al., 2012 for further details) obtained from the same subjects. Identical pre-processing and post-processing steps were applied. A schematic diagram of the work-flow used here is presented in Supplementary figure 3A. Prior to clustering, the complete dataset was evenly split into three blocks of data. The time-points (or equivalently image volumes) 1 to 400 for all subjects were placed in the first block (here named A), 401 to 800 were placed in the second block (B), and time-points 801 to 1200 was allocated to the third block (C).

For all three data blocks, a clustering using k-means algorithm was computed for k =2 to 20. In the next step, for each k, we paired the clusters between each combination of block dataset (i.e. possible parings in this case are AB, AC and BC) based on the average BOLD activity for each node in each cluster (see Supplementary Figure 3B that shows an example for k = 8). The paring was achieved by computing distance matrices between each data block's clusters using the averaged taxicab distance (see equation (1)) for the average BOLD activity of each node. This procedure yielded a k x k distance matrix for each unique paring of block dataset. Subsequently, the so called Hungarian Algorithm (Kuhn 1955; Munkres 1957) was used to find the optimal cluster pairing for each distance matrix. The Hungarian Algorithm was applied to the clustering

paring distance matrices and the output is a k x 2 solution matrix for each of the block dataset pairings. Since there are three possible block dataset pairings, it is conceivable that the optimal solutions for the different block dataset pairings are not transitive and thereby resulting in incompatible clustering solutions. This possibility was accounted for by checking if the pairing of clusters in the block dataset pairing AC was identical to the pairing of clusters that could be derived for AC by using AB and BC. After checking for transitivity among possible pairing of data blocks, nine of the tested k values had transitive solutions (k = 2, 3, 4, 5, 6, 7, 8, 11 and 12). This suggests that any choice of k that has a transitive solution is to some extent reproducible in terms of k-means clustering with similar spatial patterns independently in different parts of the second data set. Hence, by the validation of our approach using an independent data set, we believe that our choice of setting k=8 is justified, since it presented us with similar global spatial patterns for the three different parts of an independent dataset. Additionally, our key results were replicated using other choices of k (for results setting k to 5 and 12, see results shown in Supplementary Figure S7). It should be noted that our justification of choice of k that includes the splitting of an independent dataset into three parts relies on the assumption that the number of derived states in the same subjects will identical.

Supplementary Figures.

Fig. S1. An illustration of the concept of estimating the co-variance between ROI signal intensity time-courses based on non-neighboring time-points in different clusters and the creation of functional connectivity time-series. In this example, the BOLD signal intensity time courses from two seed regions located in the ACC (red line) and insula (blue line) are first extracted (A). For simplicity, we here show for a single subject and, in contrast to Figure 1, how the PBM method effects a single connection between two brain regions, rather than the entire graph. The signal intensity time-series are subsequently divided into different clusters using the k-means clustering algorithm (see Figure 1 and Methods section) which are here shown for the case of k=8 using a coloring scheme displayed above the signal intensity time-series in panel (A). In a subsequent step, the co-variance is computed for all time-points that are allocated for each cluster (B). The final step is shown in panel (C) where a functional connectivity time-series between the ACC and the insula is created based on the co-variance computed in (B) for the eight different clusters. An additional descriptive illustration that includes the clustering step is given in Figure 1 and a detailed account is given in the methods section.

Fig. S2 Regions of interest and the resting state network template chosen. 264 regions of interest (10mm spheres) was defined (Power et al. 2011) and resting state network assignments based on (Power et al., 2011 and Cole et al., 2013) for each node corresponding to the color of the text. Unclassified nodes are shown in white. Cerebellum is not shown. These regions and network assignments are used through the analysis.

Fig. S3 This figure shows a schematic plot together with example data that visualizes the strategy used to further justifying our choice of k in a separate independent dataset. (A) Shows a schematic outline of the work-flow described in the methods section. Panel (B) shows the average BOLD standardized intensity across time for all nodes and states in three different blocks (A, B and C) of the second dataset when k=8. The clusters were sorted using the Hungarian algorithm (see also for comparison Figure 2C and the methods section for further details).

Fig. S4 Euclidean distance of ROIs between two successive time points. The sequence of time points shown in the Figure are of length 8, where the first four come from one state and the latter four come from another state. Thus, at time point -3, it marks the Euclidean between the first and second time points in the sequence (both belonging to the first state). Time 0 marks the transition between states and it is the only time point where there is a difference of state membership. Time point 1 in the plot marks the difference in Euclidean distance between the first and second time point in the second state. No others state transitions occur anywhere else in the sequence. The solid line represents the averaged distance taken over all instances where the sequence could be constructed (i.e transitions between all states), pooling over all subjects and

averaged. Error bars show the standard error.

Fig. S5 The eight states/s-graphlets derived from the k-means clustering are here shown using the spring-embedded Kamada-Kawai representation. Only the top 5 percent of all edges, similar to Figure 2D, were included in the plots.

Fig. S6 No state corresponds exclusively to movement. Distribution of time-points assigned to each state at time-points for which FD>0.5, indicating high micro-movements. There is no state which is exclusively found to be associated with estimated micro-movement artifacts.

Fig. S7 Histograms showing the duration of stay in all eight states before a switch to another state occurred. The x-axis shows the duration in each state expressed in TR (TR = 0.72 s) and the y-axis show the number of occurrences for each particular duration. The number of state is shown at the top of each histogram.

Fig. S8 Replication of the burstiness of brain connectivity for different choices of k (k = 5 and 12 in the k-means algorithm) used to derive s-graphlets. Similar to the results shown in Figs. 6C-F, the percentage of edges that were significantly bursty or tonic/periodic ($p \leq 0.05$, two tailed) is shown using two different edge thresholds (withholding either the top 5 or 10 percent of all edges).

FigureS1

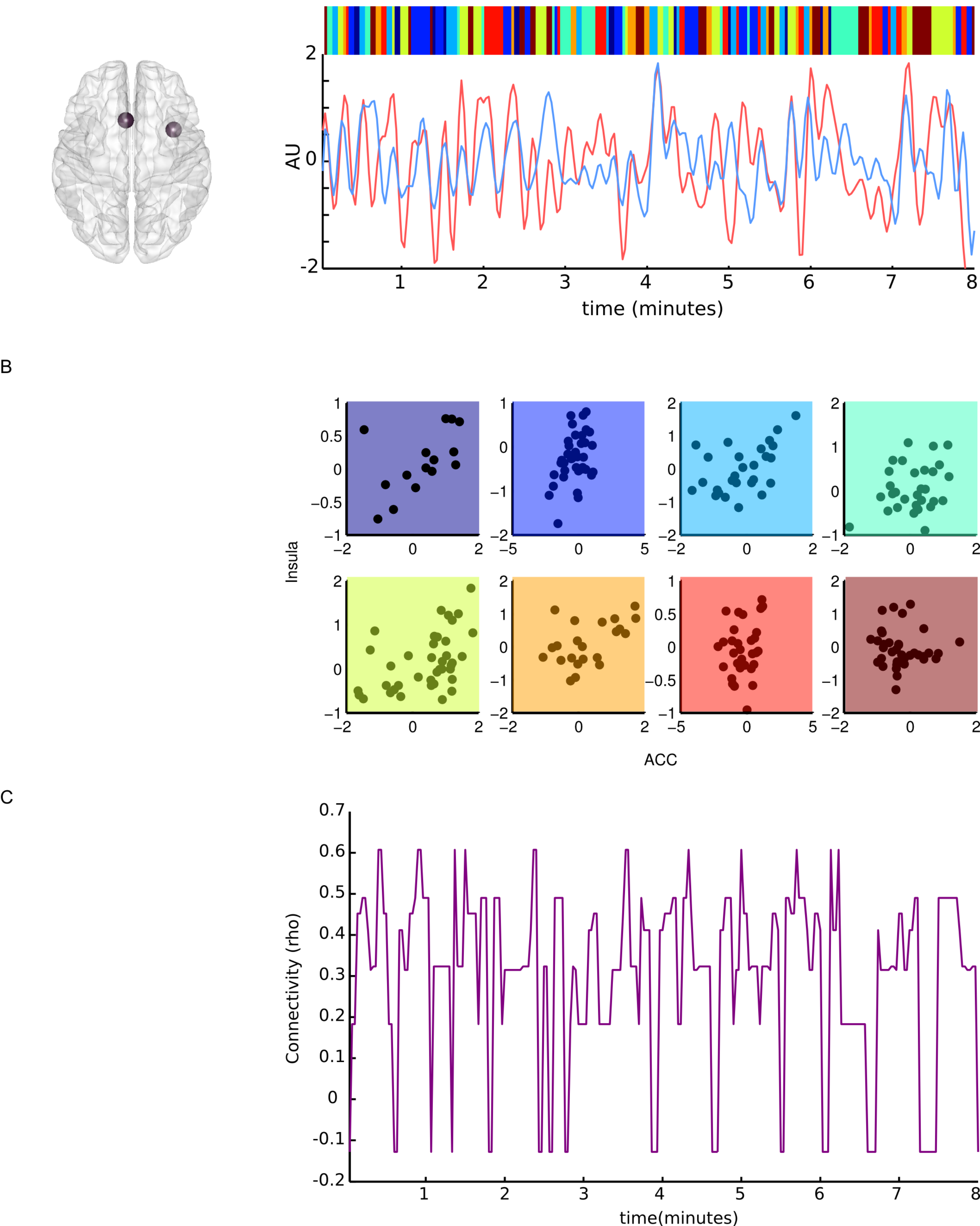
A
AU
time (minutes)
B
Insula
ACC
C
Connectivity (rho)
time(minutes)

FigureS2

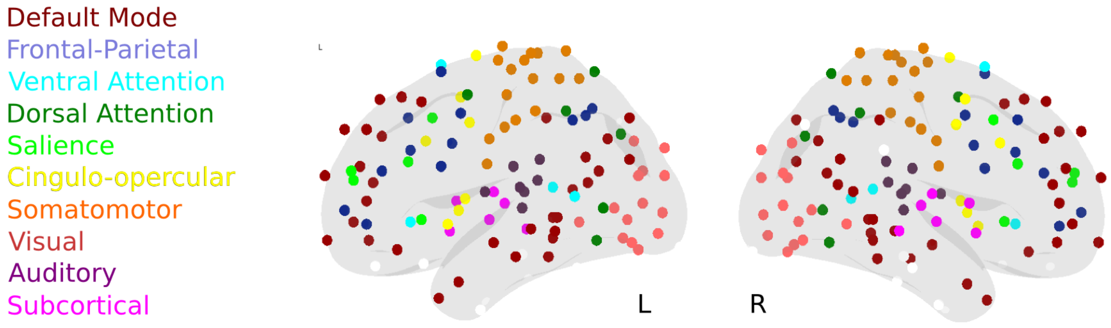
Default Mode
Frontal-Parietal
Ventral Attention
Dorsal Attention
Salience
Cingulo-opercular
Somatomotor
Visual
Auditory
Subcortical
L
R

FigureS3

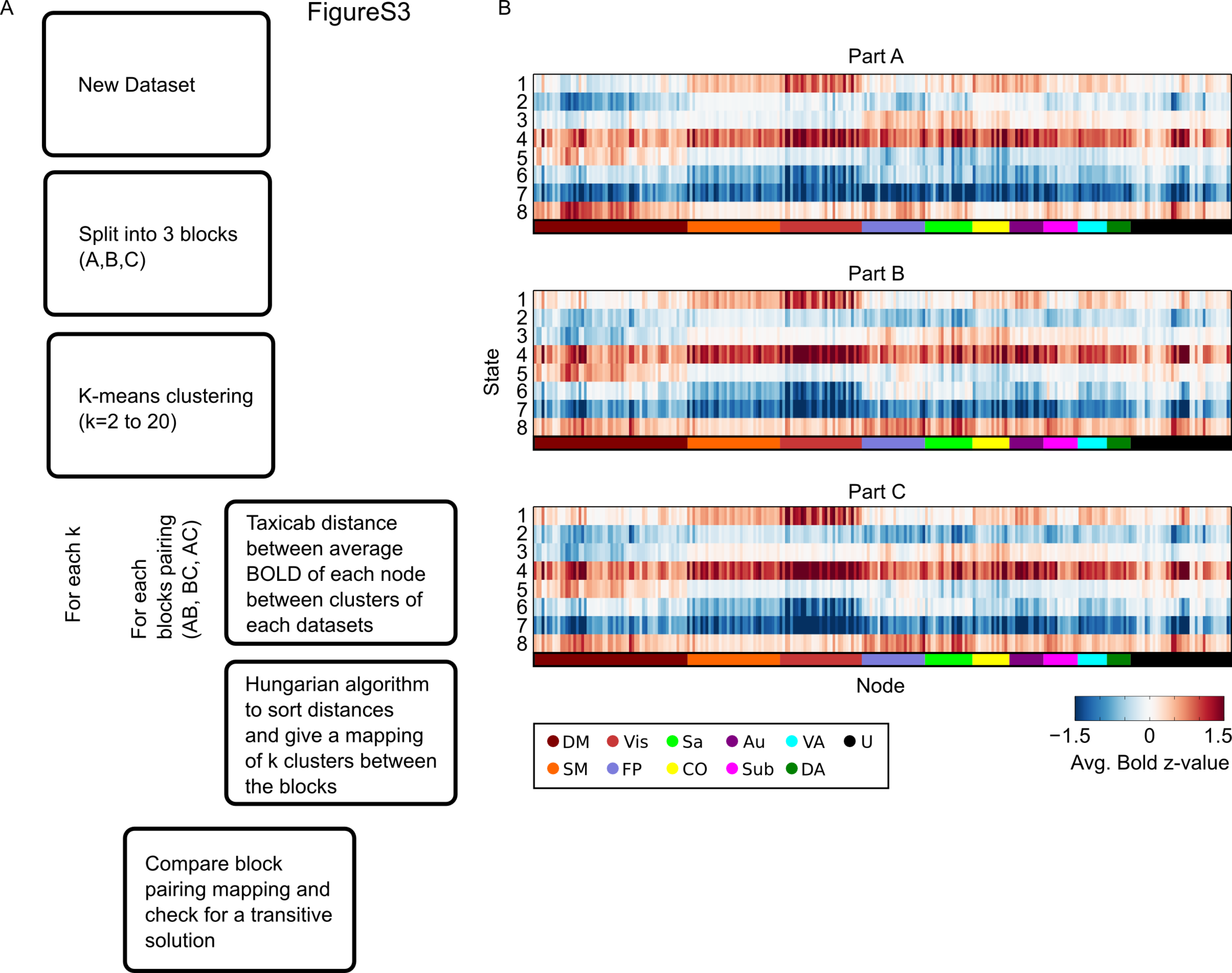
A
New Dataset
Split into 3 blocks (A,B,C)
K-means clustering (k=2 to 20)
For each k
For each blocks pairing (AB, BC, AC)
Taxicab distance between average BOLD of each node between clusters of each datasets
Hungarian algorithm to sort distances and give a mapping of k clusters between the blocks
Compare block pairing mapping and check for a transitive solution
B
Part A
Part B
Part C
State
1
2
3
4
5
6
7
8
Node
DM
Vis
Sa
Au
VA
U
SM
FP
CO
Sub
DA
−1.5
0
1.5
Avg. Bold z-value

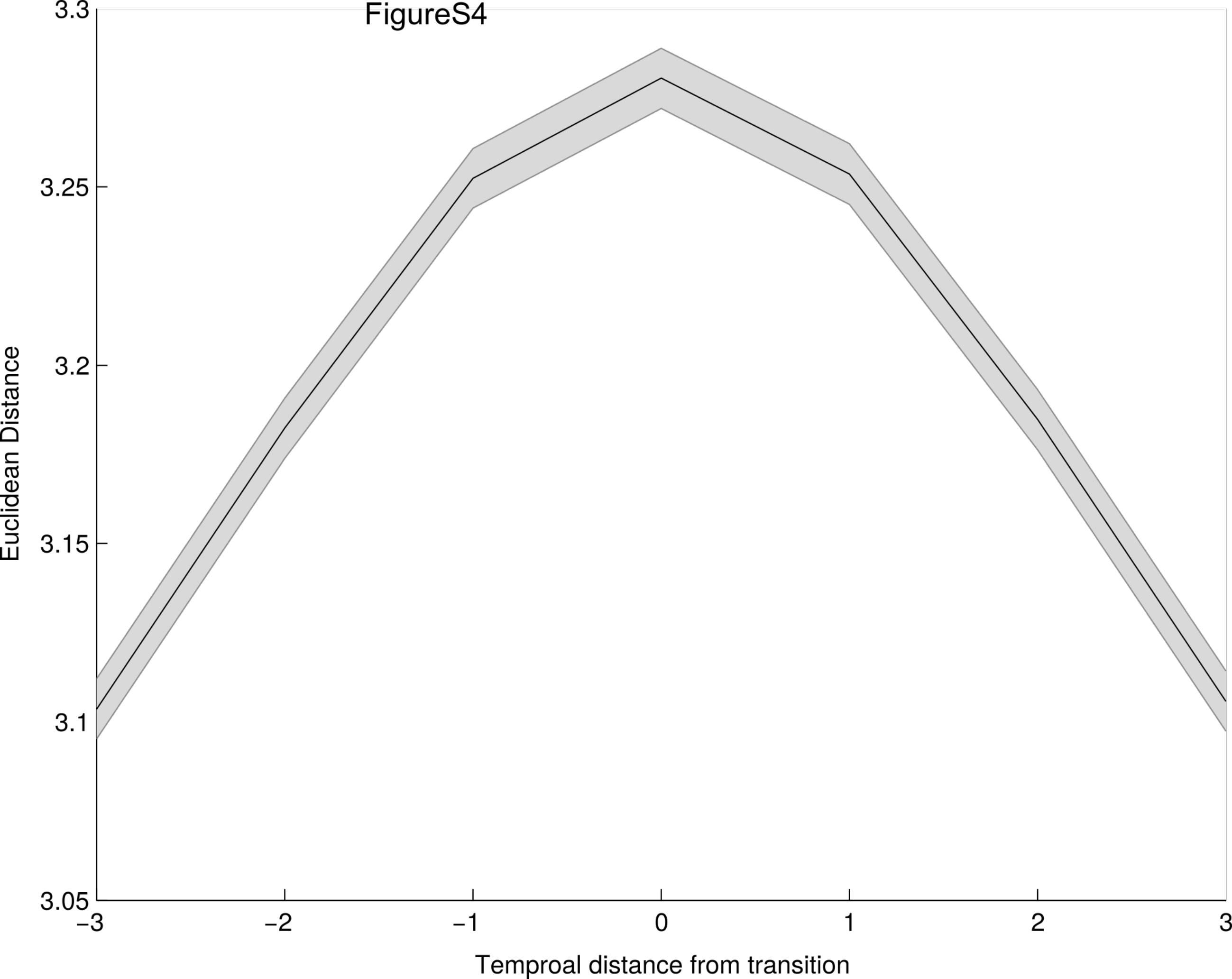
FigureS4
Euclidean Distance
3.3
3.25
3.2
3.15
3.1
3.05
−3
−2
−1
0
1
2
3
Temproal distance from transition

FigureS5

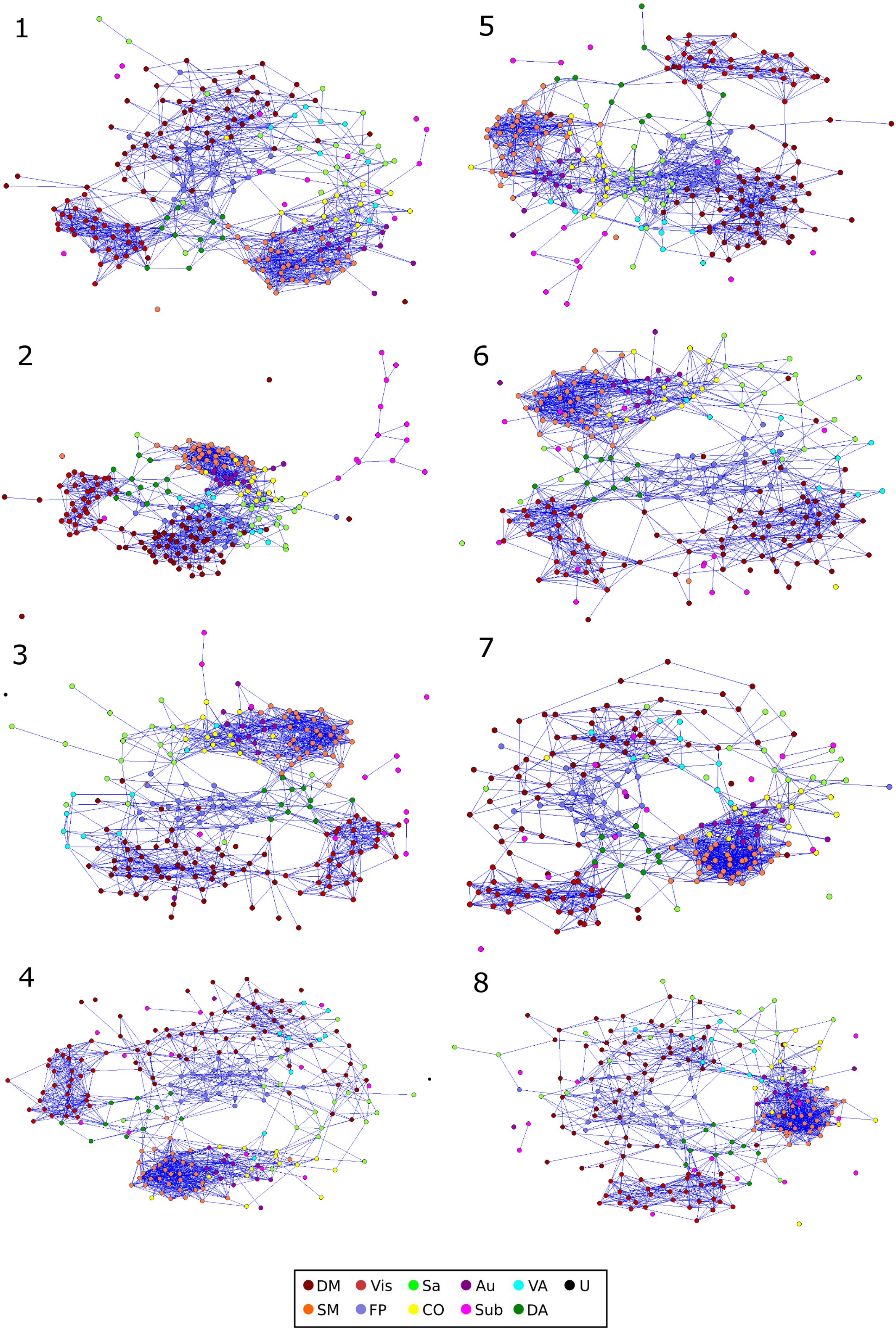

FigureS6

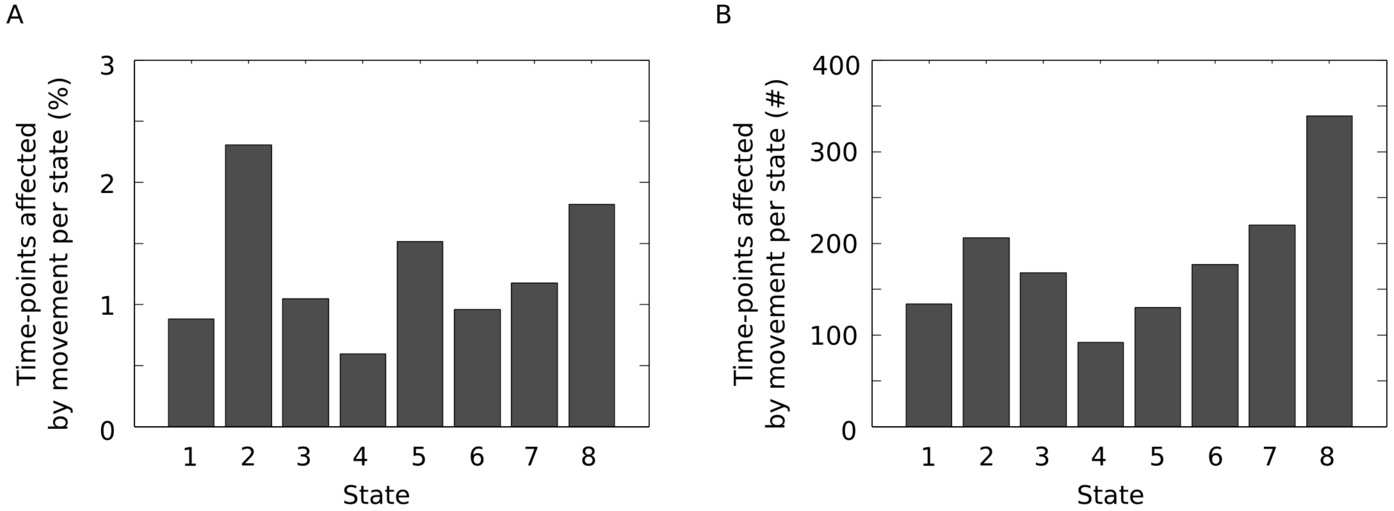
A
Time-points affected by movement per state (%)
0
1
2
3
1 2 3 4 5 6 7 8
State
B
Time-points affected by movement per state (#)
0
100
200
300
400
1 2 3 4 5 6 7 8
State

FigureS7

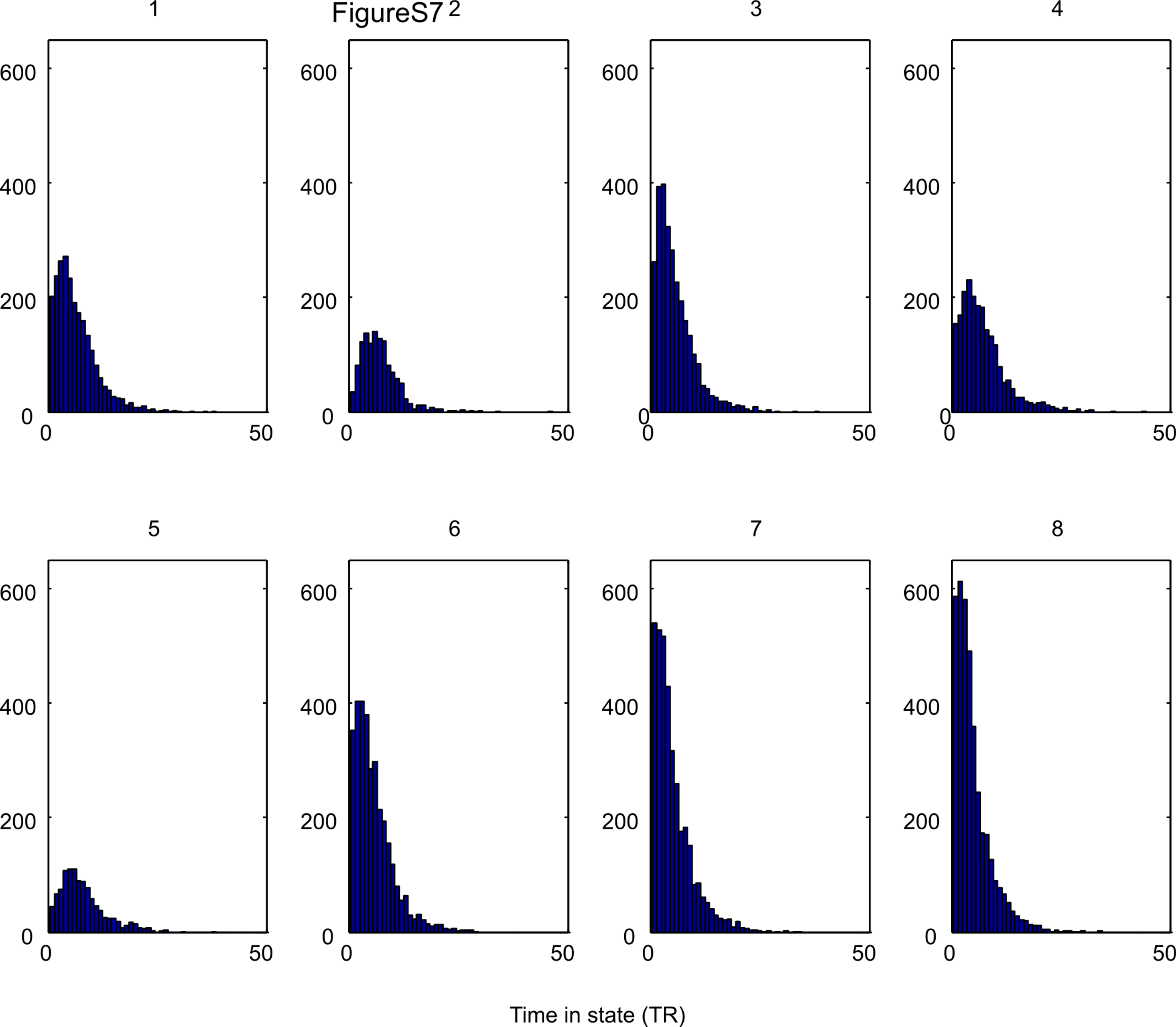

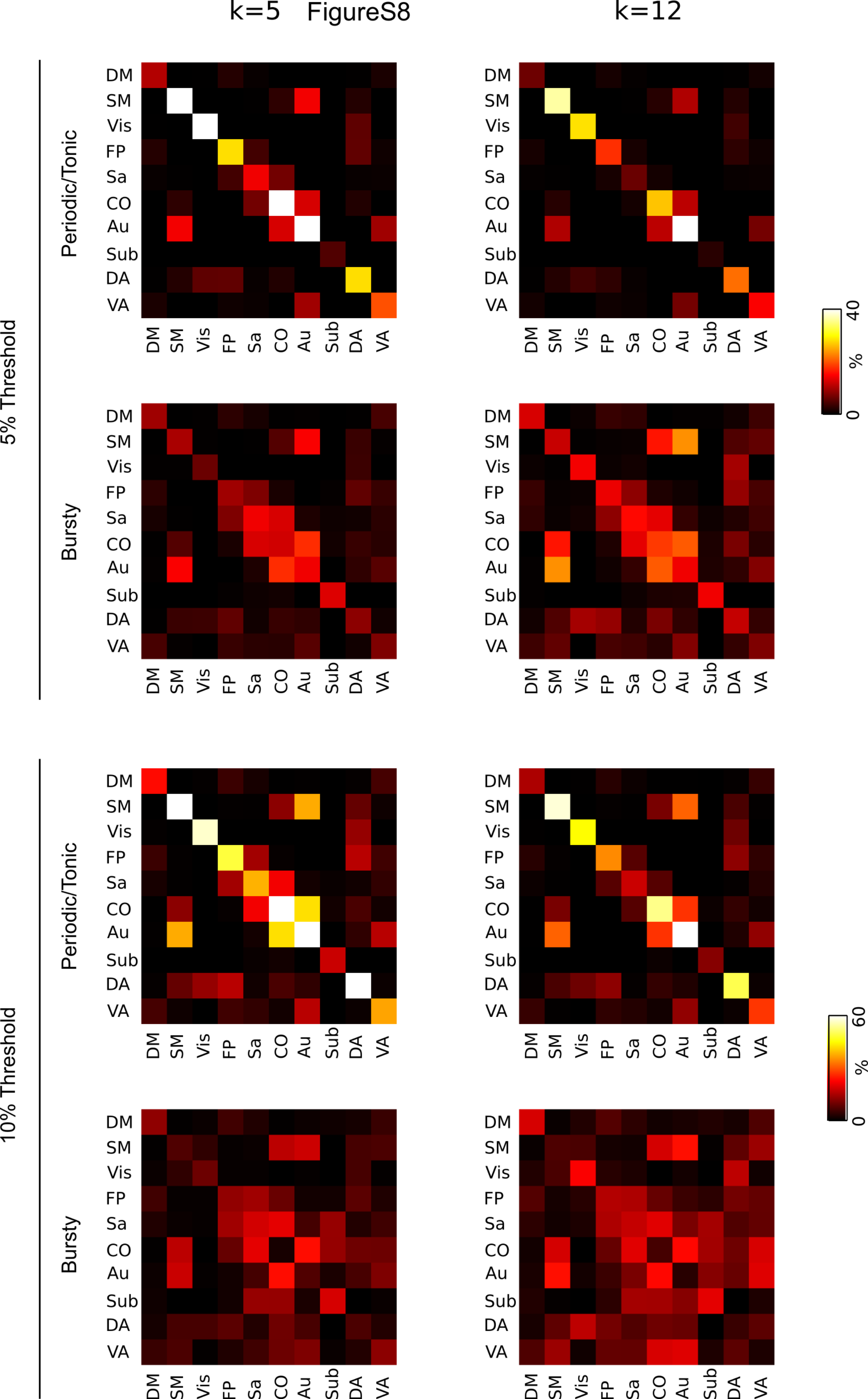
k=5
FigureS8
k=12
5% Threshold
10% Threshold
Periodic/Tonic
Bursty
DM
SM
Vis
FP
Sa
CO
Au
Sub
DA
VA
40
%
0
60
%
0
